# Disaster Health Literacy – development and validation of a short measurement instrument in German to supplement the HLS_19_ instruments

**DOI:** 10.3389/fpubh.2025.1589705

**Published:** 2025-07-25

**Authors:** Michael Ewers, Joachim Beckert, Lennert Griese, Michael Köhler, Anita Prasser, Himal Singh, Doris Schaeffer

**Affiliations:** ^1^Institute of Health and Nursing Science, Charité - Universitätsmedizin Berlin, Corporate Member of Freie Universität Berlin and Humboldt-Universität zu Berlin, Berlin, Germany; ^2^School of Public Health, Bielefeld University, Bielefeld, Germany

**Keywords:** disaster & risk management, health literacy, instrument development, measurement, content validity, face validity, disaster health literacy

## Abstract

**Introduction:**

In many countries, promoting the ability to protect one’s own life and health and to help oneself when faced with adversity is a cornerstone of disaster risk reduction. For this reason, the population must be able to access, understand, assess and apply the information necessary to do so. So far, there have been only a few attempts to define this so-called “Disaster Health Literacy” (DIS-HL), and there is a lack of easy-to-use instruments to measure it. The aim of this research was to develop such an instrument based on a conceptual framework and to report on its content and face validity.

**Methods:**

As an interdisciplinary and interinstitutional working group we applied a standardized approach to the development of instruments for social science survey research. Based on a scoping review, we constructed a conceptual framework and defined an items list. We conducted an online expert review (*n* = 12) to test content validity and used cognitive interviews (*n* = 10) and real-life interviews (*n* = 10) to assess face validity. Item and scale context validity indices were calculated, and a formal and summative analysis of the qualitative data was carried out.

**Results:**

Based on suggestions from the literature, we defined DIS-HL and identified key components of the construct. In item development, we considered four cognitive dimensions (access, understand, appraise, apply), the heterogeneous information requirements before, during and after events, and three information task domains (prevention, acute response, access to care). Based on the experts’ feedback and the results of the face validity assessment we reduced the original 15 items to 12 items. Furthermore, we made moderate adjustments to the content and language of the items and shortened the introductory text to improve clarity and comprehensibility.

**Discussion:**

Our research strongly supports the relevance and content validity of the short DIS-HL^GER^ measurement instrument. However, further psychometric tests (factor analysis) are necessary to verify its quality. To support this, a large-scale pilot test will be conducted as part of the third national representative survey on health literacy in Germany (HLS-GER 3). If this test is successful, an English translation and international adaptation of this instrument could be considered.

## 1 Introduction

Disruptions to the functioning of communities and societies are occurring with increasing frequency and severity worldwide. Depending on exposure, vulnerability and capacity, they can cause enormous environmental, material, economic and human damage. To counteract this, governmental and non-governmental organizations developed comprehensive disaster risk reduction measures in line with the Sendai Framework (1). One of these measures is to improve disaster preparedness among the general population by raising awareness and disseminating information on how individuals, families and communities can become more resilient. In Germany, for example, risk communication by the authorities and self-protection of the population are a cornerstone of the National Resilience Strategy, which, however, was only published in 2022 and has yet to be implemented in large parts (2). At the international level, there are already several initiatives in place to increase the population’s ability to prepare for potential hazards, to behave responsibly and to protect themselves in public health emergencies, acute crisis and disaster situations (3, 4). Furthermore, they intend to enable the people to make contributions to minimizing the consequences of disasters, and to work together with their communities to restore some degree of normality.

A prerequisite for the effectiveness of these measures is that quality-assured information on disaster risks and mitigation strategies is publicly available, and that the population can use this information for their own self-protection and self-help. However, there are several limiting factors to consider on both the provider and recipient side of the information. One important factor on side of the recipients is the large number of people with poor reading and writing skills, low numeracy or illiteracy, even in developed countries (5), which is likely to have a negative impact on the ability to handle disaster-related information. According to Article 11 of the UN Convention on the Rights of Persons with Disabilities (6) special attention should be paid to the inclusion of these and other at-risk populations in disaster risk reduction measures (7). From a public health perspective, in this context, some experts emphasize not only the importance of functional literacy and numeracy, but also the need to promote a specific disaster-related health literacy in order to be successful in disaster risk reduction, response and recovery (8–10).

In the meantime, there have been a few attempts to define and assess this specific form of literacy (11). The priorities set in these attempts vary greatly, as do the scope and scientific quality. Some authors focus on preparing for disasters or dealing with individual risks in general (12–14). Others single out specific aspects, including protecting one’s own life, and personal safety (15–17). Only few authors explicitly refer to international efforts to define and measure health literacy and apply these to the disaster discourse (18). The HLS-EU Consortium defines health literacy as the *“knowledge, motivation and competences, to access, understand, appraise, and apply health information in order to make judgments and take decisions in everyday life concerning health care, disease prevention and health promotion to maintain or improve quality of life during the life course”* (19) (p. 3). This generic definition does not explicitly mention disaster-related health risks, or how to handle information that is necessary for self-protection and self-help when faced with adversity. This could make it difficult to assess and monitor this specific form of health literacy and thus to take targeted disaster risk reduction measures (8, 10).

The available measurements tools, based on the above-mentioned definition, such as the HLS_19_-Q47, and two adapted short forms, HLS_19_-Q16 and HLS_19_-Q12, are validated for monitoring general health literacy (20, 21). Over the last years, some supplementary packages have been developed to cover specific topics such as Digital Health Literacy [HLS_19_-DIGI (22)], Navigational Health Literacy [HLS_19_-NAV (23)], and Communicative Health Literacy [HLS_19_-COM (24)]. This includes efforts to define and measure a form of health literacy specifically geared to the COVID-19 pandemic (25–27). This pandemic related research concluded, among other things, that there is an urgent need to better prepare the population to deal with information in future public health emergencies. However, the general dimensions of health literacy in line with the all-hazards approach to disaster risk reduction (28, 29) were not considered in these activities. This is why there is still a lack of validated and easy-to-use instruments to assess and monitor disaster-related health literacy. To fill this gap, this research aims to develop and validate such a supplement to the HLS_19_ measurement instruments, initially in German language.

## 2 Materials and methods

We set up an interdisciplinary and interinstitutional working group, consisting of German members of the WHO Action Network on Measuring Population and Organizational Health Literacy (M-POHL) on the one hand, and public health and disaster nursing experts, on the other. This working group was co-chaired by the first and last author. For our research, we decided to apply a standard methodological approach for the development of instruments for social science survey research (30). The first part of the instrument development process took 9 month (April to November 2024) and consisted of four steps (S1-S4), which are documented in Table 1 and explained in detail below. The second part of the development process, the subsequent piloting of the new instrument and the application of exploratory and confirmatory factor analyses will be documented in a separate publication.

**Table 1:** Methodological approach to the development of the DIS-HL^GER^ instrument.

Step	Objectives	Measures
S1	Conducting a scoping review	Literature review with 124 articles in full-text analysis and 26 publications ultimately included; narrative summary of the results of the review
S2	Constructing a conceptual framework	Definition of disaster health literacy and concept mapping process in the research team leading to a conceptual framework
S3	Defining and consenting items	Generating an initial item pool based on disaster-related information tasks (36 items), step-by-step reduction to 15 items in team discussion rounds (relevance, significance, comprehensibility etc.)
S4	Testing content validity and face validity	Online expert review (*n* = 12), cognitive interviews (*n* = 10) and real-life interviews (*n* = 10); calculation of I-CVIs and S-CVI; formal and summative analysis; continuous revision, reduction and modification of the item list and introductory text

### 2.1 Conducting a literature review

In a first step (S1), we conducted a scoping review (31), which is an ideal approach for summarizing the breadth of the existent knowledge base on the topic of interest here. With this literature review we aimed to answer the following questions:

*Are there approaches in the literature for defining and measuring disaster-related literacy at the population level? What is the significance of health dimensions or health problems in this context? How are existing definitions, concepts or instruments of disaster-related literacy linked to the HLS_19_ measurement instruments?*

We searched the databases MEDLINE, CINAHL, PsycINFO and SocINDEX and DACH-Information via EBSCOhost, as well as the Cochrane Library-Database from April to May 2024. This search was extended by a hand search in the lists of references of texts already found in key journals, and on websites of relevant disaster risk reduction organizations. As search terms we used “disaster risk reduction,” “disaster literacy,” “health literacy” describing the concept of health literacy combined with search terms describing the context of disasters like “emergenc*,” “cris*,” “disaster*,” “public health emergency.” Since the topic of disasters has received significantly more attention since the terror attacks of 11 September 2001, we included literature published between 2001 and the time of the search. There were no restrictions on language, publication status or quality. The inclusion and exclusion criteria were based on the PCC scheme (Population, Concept, Context) with a focus on publications dealing with conceptual or measurement issues in context of literacy, health and (public health) emergencies, crisis or disasters of any kind. Studies that use the concept of health literacy (1) in general, (2) as part of a definition, (3) for an instrument or (4) develop a concept themselves were included. If the focus was exclusively on (intentional) misinformation, if only organizational perspectives or only technical aspects of disasters were discussed, if an intervention or technology was described or if no health aspects were considered, the publications were excluded. Conference papers, letters, blogs, press releases and theses were disregarded (see Table 2 for inclusion and exclusion criteria).

**Table 2:** Inclusion and exclusion criteria (scoping review).

Domain	Inclusion criteria	Exclusion criteria
Population	No limitations	
Concept	Health literacy	
	Instruments/Measuring Providing a definition Proving a concept Using the concept	Focusing on misinformation only and not on accessing, understanding, appraising, applying information Professionals/organizational perspective Technology and intervention Not health related concepts/definitions
Context	Disaster	
	Public Health Emergency Crisis Man-made	If it is not a public health emergency
Years	2001–2024	
Language	No limitations	
Publication status	No limitations	
Sources		Conference papers, letters, blogs, press release, theses

**Figure 1:**
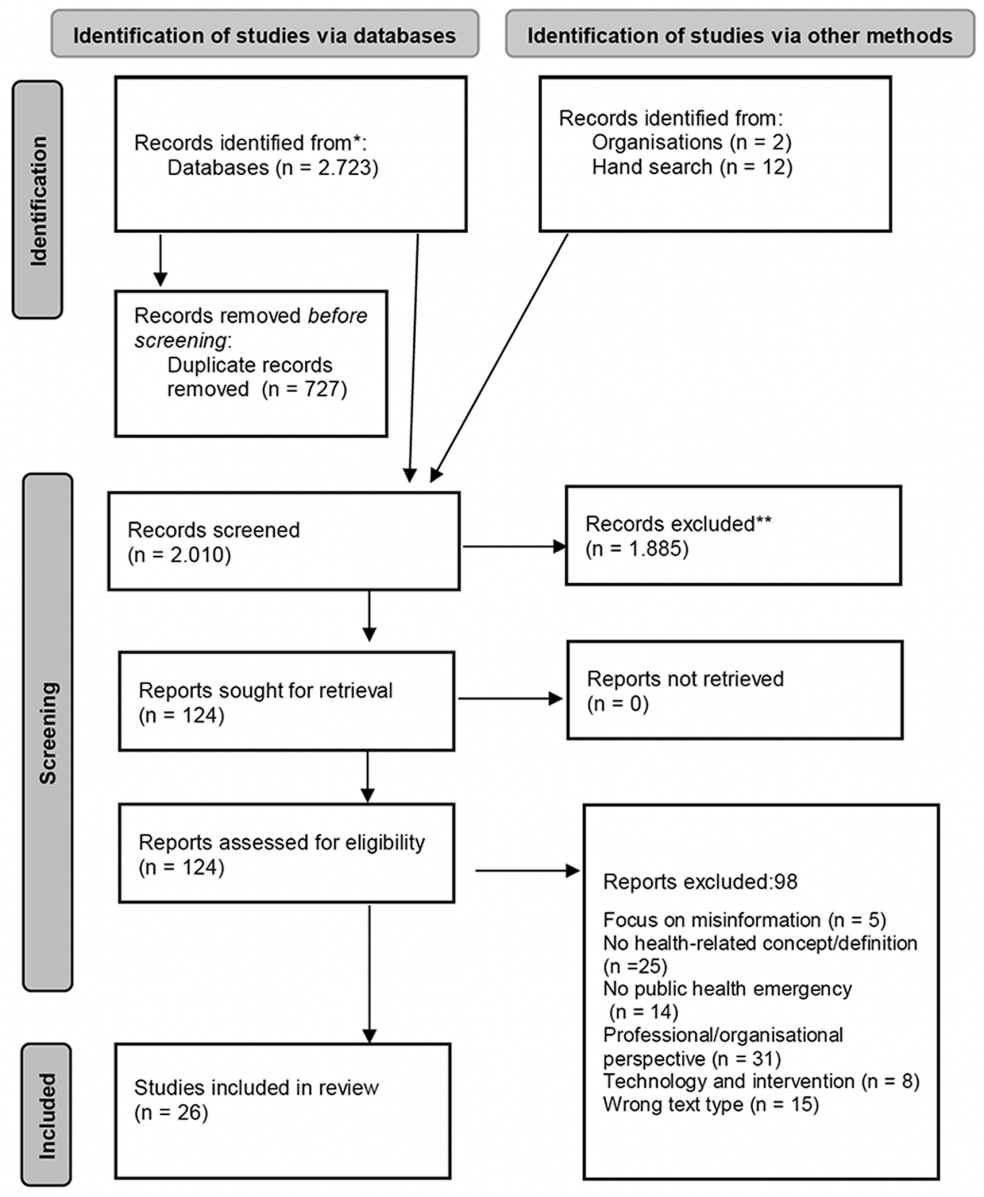
PRISMA flow diagram of the scoping review process.

The literature search identified 2,723 publications out of which 727 were duplicates. Two independent reviewers (JB, AP) screened the titles and abstracts of the remaining 1,996 publications. 1,885 publications were excluded because they often referred to disasters with no apparent health implications or were devoted to the dissemination of information between disaster control organizations. 124 publications were subjected to a full-text review. Further publications were then sorted out, partly because they did not contain a health-related or health literacy-related concept or definition, did not deal with public health emergencies, contained only organizational perspectives or expert opinions, or focused on misinformation or the more technical aspects of disasters. Conflicts between the two reviewers were resolved by consulting a third reviewer (MK). Two researchers (JB, AP) analyzed the 26 publications finally included, extracted the data relevant to the research question and created a narrative summary. The entire procedure is documented in a PRISMA flow diagram (32) in Figure 1.

### 2.2 Constructing a conceptual framework

**Figure 2:**
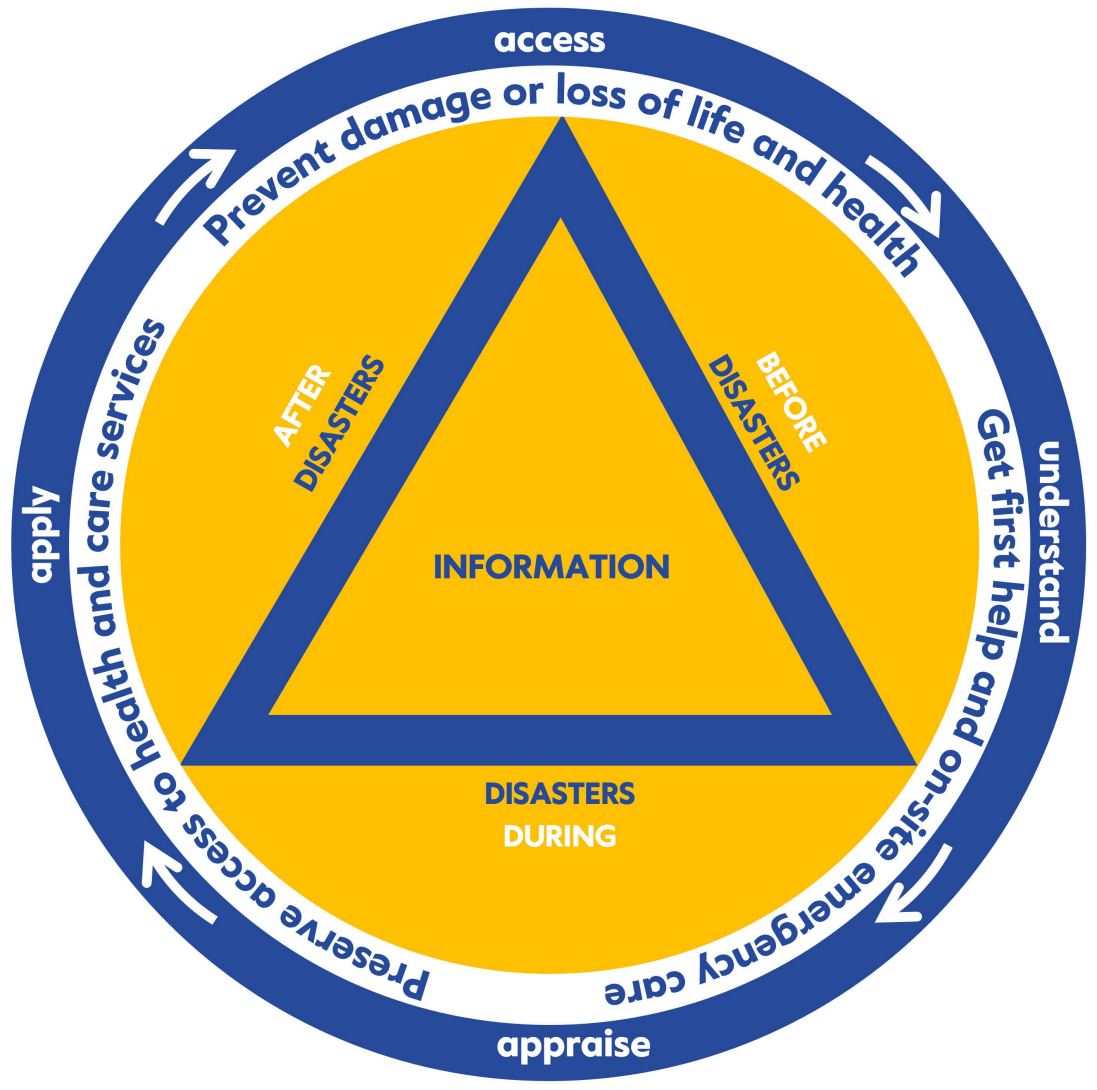
Conceptual framework for measuring DIS-HL (self-developed model).

In a second step (S2), we critically reflected on the results of the scoping review and developed a working definition for “Disaster Health Literacy” (DIS-HL), inspired by several suggestions from the literature and previous work on disaster risk reduction, disaster literacy, and health literacy. In a total of eight discussion rounds, we worked out a conceptual framework for the development of the DIS-HL instrument, considering the four cognitive dimensions of health literacy (access, understand, appraise, apply), the heterogeneous information requirements during the disaster cycle (before, during and after disasters), and three task domains (prevention, access to care, acute response). To this end, we searched the literature for specific information tasks that people must cope with in terms of their health-related self-protection and self-help when confronted with adversity. In cases where the literature remained vague, members of the research team with disaster (nursing) expertise processed and incorporated experiences from previous events or used brochures from disaster relief organizations for inspiration (33). This step served to define the key components, dimensions and facets of the construct to be assessed with the instrument. The conceptual considerations were then condensed and visualized graphically (see Figure 2).

### 2.3 Defining and consenting items

In a third step (S3), we operationalized the previously developed concept and defined 36 items to make DIS-HL measurable. Where possible, we draw inspiration from existing instruments on disaster literacy or COVID-19 related literacy tools from the literature (25–27, 34). However, this was only possible to a limited extent. For later compatibility, we modeled the intended length, the wording of the items, and the response scale based on those of the existing supplementary packages for the HLS_19_ questionnaire (22–24). As the project progressed, we reduced the original item pool in the authoring group. This reduction was informed by various factors, including aspects of practicability, representativeness, everyday relevance, specificity for health topics, and linguistics. The objective of this reduction was to eliminate items that were poorly formulated or ambiguous by means. In addition, we decided which items were best suited to cover the relevant aspects of the conceptual construct of DIS-HL. This process resulted in the retention of 15 items, which were then subjected to the following validation process (35).

### 2.4 Testing content validity and face validity

In a fourth step (S4), we conducted an online expert review to assess how well the developed instrument covers all relevant parts of the DIS-HL construct. In addition, we conducted cognitive interviews and real-life interviews to verify that the instrument appears to measure what it is supposed to measure and to obtain feedback on the clarity, comprehension and interpretability. For pragmatic reasons, the expert review and cognitive interviews were carried out in parallel. In both cases, identical lists of 15 items were used. The further reductions or changes were subsequently made based on these results and intensive discussions within the research team. This resulted in a reduced 12-item list which was then used for the real-life interviews.

For the online review, we recruited experts who had expertise either in health literacy (*n* = 6), disaster risk management (*n* = 5) or research methodology (*n* = 1). The experts got access to the DIS-HL instrument via the online tool SoSci Survey (36) and were asked to rate each item on a four-point-Likert scale (1 = not relevant, 2 = somewhat relevant, 3 = fairly relevant and 4 = very relevant). Furthermore, we invited the experts to provide commentary or propose modifications to the items or the introductory text, or to suggest topics that may have been overlooked. We calculated a content validity index for each item (I-CVI) and the whole scale (S-CVI), numerical measures widely used to indicate the proportion of experts who believe that an item is relevant or representative of the content area (37). The S-CVI was calculated as sum of the ratings 3 and 4 divided by the total number of experts (*n* = 12) divided by the number of items. Results ≥ 0.8 were considered favorable. The qualitative feedback from the experts was formally analyzed and used for the instrument revision.

Two members of the research team (AP, JB) conducted face-to-face cognitive interviews (*n* = 10) using a semi-structured interview guide with probing techniques (38). We recruited participants via convenience sampling, trying to ensure variation in key socio-demographic characteristics. The age of the participants ranged between 24 and 86 years, with an equal number of women and men. The socio-economic status (SES) of participants ranged between 3 and 8 on the MacArthur subjective SES Scale (39), their educational level ranged from 3 to 7 according to the ISCED Standard Classification of Education (40). Written and informed consent to voluntary take part in the study was obtained from all participants. The interviews were transcribed and extracted in a case-specific listing for formal analysis (41). In the event of ambiguities or misunderstandings, item-specific recommendations were made to alleviate their effects and optimize the comprehensibility of the items.

In addition, real-life interviews were conducted by researchers (*n* = 10) with extensive experience in survey research from the Institute for Population Research Allensbach (IFD Allensbach). They used a revised and modified 12-item list as supplement to the HLS19-Q47 in October 2024 in a pre-test for the third national representative survey on health literacy in Germany (HLS-GER 3). Emphasis was placed on comprehensibility of the instrument and questionnaire experience in this context. The subjective assessment of the interviewers’ experiences was put through a summative analysis, which was used to finalise the DIS-HL instrument.

## 3 Results

### 3.1 Scoping review

The analysis of the 26 publications included in our review shows that the way the population deals with potentially life-saving information in the context of disasters is receiving increasing attention. This applies particularly to countries and regions that have been more frequently or more intensely affected by disasters in the past, like North America (42–45), the Asia-Pacific region (46–50), or the Middle East (51–54), especially Turkey (11, 34, 54). While publications from these world regions cover a wide range of disasters, including the COVID-19 pandemic (43, 49, 55), it is primarily this event that has sparked particular interest in the topic in several European countries (25–27, 56, 57).

Most of the publications included are conceptually oriented and aim to contribute to improving crisis communication in the context of disaster risk reduction. The terms chosen for this topic range from “Disaster Literacy” over “Disaster Prevention Literacy,” “Disaster Risk Literacy” and “Disaster Preparedness Literacy” to “Disaster Health Literacy” (11, 18). In some cases, particular types of disasters are addressed, such as in “Natural Disaster Literacy” (52), “Flood Preparedness Literacy” (48) or “Covid-19 Health Literacy” (19); in other cases, specific cultural factors are considered, as in the case of Japan where traditional “Bousai Literacy” and “Gensai Literacy” are presented as two forms of “Disaster Risk Reduction Literacy” (25). Some of these various definitions are already documented in a previous systematic review on this topic (11).

One underlying premise in many of the included publications is that the population must be able to handle specific information to ensure their own safety, to collaborate with the emergency services or disaster relief teams, and to avoid or minimize losses of any kind in the various phases of the disaster cycle (42, 44, 47–50). Even if it is not always clear from the terms and definitions chosen, health dimensions play a more or less important role in all the concepts mentioned above. In some cases, securing one’s own survival or providing basic emergency care is treated at the same level as securing the material basis for one’s livelihood (47, 48, 58). Yet in other cases, the protection of life, health and well-being and therefore the public health dimension of disasters is the focus (11, 18, 49, 56). While some authors look at the general population or randomly selected population groups, others draw attention to sub-populations who live with functional and health needs or who have problems to access care (42, 44).

In their frequently cited work, Brown et al. (8) criticize the lack of alignment of disaster-related information processing with the reading and writing abilities of at-risk populations, particularly the older adult. They define “Disaster Literacy” as *“an individual’s ability to read, understand, and use information to make informed decisions and follow instructions in the context of mitigating, preparing for, responding to, and recovering from a disaster”* (8) (p. 267). They developed a conceptual model with reference to the US discourse on health literacy, in particular to authors such as Paasche-Orlow/Wolf (59) and Nutbeam (60). This model considers several predisposing factors, four levels of literacy (basic, functional, communicative/interactive, critical) and various patient- and environment-related factors. The application of this model should help to better align disaster-related information with the literacy level of at-risk populations (10). However, they have not developed a disaster literacy measurement tool based on this model.

By contrast, Çali¸skan et al. (11) defined “Disaster Literacy” based on a systematic literature review as an “*individuals’ capacity to access, understand, appraise, and apply disaster information to make informed decisions and to follow instructions in everyday life concerning mitigating/prevention, preparing, responding, and recovering/rehabilitation from a disaster in order to maintain or improve quality of life during the life course*” (34) (p. 2). Based on this definition they developed a complex 16-Matrix integrative conceptual model and a self-report measurement tool for Turkey, the so-called “Disaster Literacy Scale” (DLS). This tried-and-tested instrument has 61 items and has, to our knowledge, so far been used with different populations but only in this specific cultural context (34, 55, 56). In terms of content, the “Disaster Literacy Scale” is a thematically generic instrument that covers various facets of disaster-related information. However, despite a reference to the definition from the HLS-EU Consortium (19), health dimensions play a rather subordinate role in the instrument. Other instruments mentioned in the literature, like the “Household Disaster Preparedness Index” (53), either deal with more general disaster precautions, are only available in the respective local language, or are insufficiently documented or have no clear link to the European concept of health literacy, such as the “Disaster Health Literacy Mitigation and Preparedness Questionnaire (DHLQ)” (14, 15).

Only a few authors focus directly on the health aspects of disaster-related information processing. Seifi et al., for example, define “Disaster Health Literacy” as *“the ability to critically question health-related information, the health care system in general and then use this information to actively address the social, economic and environmental determinants of health”* (51) (p. 151). They combine developmental challenges (menopause crisis) with disasters (situational crisis) and with a strong focus on the accompanied educational and information needs of aging women as an at risk-population. However, they did not develop a tool to assess “Disaster Health Literacy” with such a focus. The same applies to Sørensen who defines “Disaster Health Literacy” simply as *“the ability to access, understand, appraise, and apply information to manage health concerns in a disaster (…)”* (18) (p. 27). This short definition does not provide any further explanation of disasters, their phases or their character. In this regard, the disaster specific information needs and thus the difference between general health literacy and “Disaster Health Literacy” remains vague. Furthermore, there are no activities to make this specific form of health literacy measurable in combination with the existing HLS_19_ instruments.

In summary, it can be stated that there are already some definitions and models for disaster-related health literacy in the literature, and in a few cases, measuring instruments have been revised or developed. The existing definitions, concepts and measurement instruments for “Disaster Literacy” emphasize general disaster-related information tasks, whereas they tend to underestimate health-related dimensions. Also, the needs of people with functional and access needs are not always sufficiently addressed. The definitions of “Disaster Health Literacy,” on the other hand, only marginally refer to disaster-specific dimensions. Easy-to-use measuring instruments for DIS-HL that are compatible with the disaster risk reduction discourse and the HLS_19_ instruments are not yet available.

### 3.2 Conceptual framework for measuring DIS-HL

We used the results of the scoping review, and particularly the short definition proposed by Sørensen (18) as a starting point for developing a conceptual framework for measuring DIS-HL. To be compatible with the HLS-EU consortium’s understanding of health literacy and the HLS_19_ instruments on the one hand, and to consider aspects that are relevant for disaster risk reduction in line with the Sendai Framework (1) and the all-hazards approach (28) on the other, we decided to expand the definition as follows:

*“Disaster Health Literacy is the ability to access, understand, appraise and apply information to protect one’s own life and health or to manage health problems before, during or after a disaster of any kind.”*

With this definition, we cover the full spectrum of disasters, from extreme weather events such as heat waves or flooding to technical malfunctions, cyber-attacks or other violent events, to health disasters resulting from chemical, biological, radiological or nuclear (CBRN) events – not only pandemics like COVID-19. We highlight the relevance of individual, family, and community disaster preparedness on the one hand (9) and the cyclical nature of disasters and of the measures to respond to them on the other (1, 2). Inspired by Brown et al. (8, 42), the various phases of the disaster cycle were integrated in this definition in a simplified form. The areas of prevention, mitigation and preparedness were summarized as “before,” the response in the acute phase as “during” and rehabilitation and recovery as “after” disasters. This simplification was necessary particularly for the comprehensibility and measurability of the DIS-HL measurement tool. Although the information provided before, during and after disasters could probably also be used to protect the life and health of relatives, neighbors or other people in the near vicinity, we wanted to emphasize the idea of self-protection and self-help. The management of health problems was added to include the situation of people with functional and access needs (6, 9), not just people without any health conditions, the so-called normal population (“Normalbevölkerung”), which in Germany, despite all demographic, epidemiological and cultural changes in the last decades, is still regarded as the benchmark for disaster risk reduction measures.

We summarized the results of the mapping procedure in Figure 2. The overall design of the model is based on the international civil defense symbol used to protect civil defense organizations, their personnel, buildings and equipment or to protect civil defense structures (an equilateral blue triangle on an orange background). In the original, the Federal Office of Civil Protection and Disaster Assistance (BBK) administers the protection rights for German civil defense organizations. The outer ring shows the four cognitive steps in dealing with (health) information, which form the core of the health literacy concept. The following ring then contains information tasks summarized in three groups, which are necessary for self-protection and self-help in the context of disasters: (1) prevent damage of loss of life and health (prevention); (2) get first help and on-site emergency care (acute response), (3) preserve access to health and care services (access to care). These parts of the concept are based on the analysis of the most relevant information tasks that the population must cope within the context of disasters. In addition to enabling self-protection and self-help, they are also geared toward working with emergency and disaster relief services. They consider the numerous actual or only imagined access barriers that may need to be overcome when seeking outside help (e.g., in the case of mental health problems following a disaster). The three summarized phases of the disaster cycle are shown within the circles (before, during and after the disaster), they frame the triangle that symbolizes the disaster-related information. It is important to note that the various dimensions of DIS-HL included in the model are in a dynamic interaction with each other and with the respective environmental conditions, particularly the disaster-related (health) information and its quality.

### 3.3 Item development and content validation

Based on these conceptual considerations, 36 items were initially developed that related to specific tasks in information processing before, during and after disasters. For example, one of the information tasks in the pre-disaster phase most discussed in the research team was how to prepare an emergency backpack for possible evacuations and what should be included in it (33, 34, 53, 58). Some items referred to information about available resources in the local area that could be helpful in the event of a disaster, for example, to find shelter or fresh water to avoid infections (8, 45, 48). Other items pointed to information on how to provide first aid (34, 44) or how to manage acute health problems or existing illnesses during a disaster without immediate professional support (45, 58). The way in which information is handled concerning access to resources during a disaster (e.g., personal protective equipment, medication, aids) was also considered relevant (8, 15, 25–27). Finally, the way in which information on how to deal with trauma, shock or mental health issues after a crisis is handled was considered important (8, 18, 26, 34). The 36 items initially developed were discussed back and forth and then reduced to 15. Some items were discarded due to duplication, others were classified as not relevant or significant enough or misleadingly formulated. The list of items was preceded by a short introductory text. A four-point Likert scale was chosen as the response option, with answer categories ranging from “very easy,” to “easy” and “difficult” to “very difficult.” In accordance with the HLS_19_ instrument, this should ultimately enable the calculation of an index value (21, 61).

The expert review of the 15 item DIS-HL instrument has revealed a mixed picture. The S-CVI reached a value of 0.70 and thus had to be considered insufficient. Items 7, 9 and 15 achieved a I-CVI of 0.58 or less, items 4 and 5 had the highest ratings with an I-CVI of 0.83 and 0.94, respectively. The results of all 15 items are summarized in Table 3. The experts gave us a lot of additional comments and valuable qualitative feedback that we were able to use for the instrument revision process. This was also the case based on the cognitive interviews for the face validity, in which most items were interpreted as intended. People with a low level of education reported that the way the questions were asked was too complicated and sometimes too long. However, this is essentially predetermined by the questioning logic of the HLS_19_ instruments and could not be changed. Other respondents occasionally had comprehension difficulties especially with the “apply” items. Some of the respondents stated that the distinction between “crisis” and “disaster” was unclear to them when used together in the same item, others interpreted the German terms “Hilfsmittel” (aids) and “Notfallrucksack” (emergency backpack) in different ways.

**Table 3:** DIS-HL^GER^ measurement tool – documentation of the development process.

Final introductory text (in English)	Final introductory text (in German)
We would now like to address the topic of disasters. Disasters can occur suddenly or develop over a longer period. They significantly disrupt our daily lives and routines and can threaten or harm the lives, livelihoods or health of many people. We want to find out how easy or difficult it is to find out how to protect your life and health before, during or after a disaster and how to deal with health problems. It does not matter whether it is about you or others.	Nun möchten wir noch das Thema Katastrophen ansprechen. Katastrophen können plötzlich auftreten oder sich über längere Zeit entwickeln. Sie stören unseren Alltag und seine Routinen erheblich und können das Leben, die Existenzbedingungen oder die Gesundheit vieler Menschen bedrohen oder schädigen. Wir wollen erfahren, wie einfach oder schwierig es ist, sich darüber zu informieren, wie man sein Leben und seine Gesundheit vor, während oder nach Katastrophen schützt und wie man dabei mit gesundheitlichen Problemen umgeht. Dabei spielt es keine Rolle, ob es um Sie oder um andere geht.

Item list before content validity and face validity test (15 items) in English	I-CVI	Revisions made	Revised item list after content validity, face validity, usability test (12 items) in English	Revised item list after content validity, face validity, usability test (12 items) in German	Cognitive steps	Disaster phase	Task domains
*On a scale from very easy to very difficult, how easy or difficult is it for you to…*			*On a scale from very easy to very difficult, how easy or difficult is it for you to…*	*Auf einer Skala von sehr einfach bis sehr schwierig, wie einfach oder schwierig ist es für Sie, …*			
(01) find information on how to protect your life and health in crises and disasters?	0.75	Crisis was deleted	(01) find information about how to protect your life and health in the event of a disaster?	(01) informationen darüber zu finden, wie Sie Ihr Leben und Ihre Gesundheit im Fall einer Katastrophe schützen können?	Access	Before	Prevention
(02) find out how you can receive health warnings from safety authorities and civil protection organizations (e.g., regarding extreme weather events)?	0.75	Clarification of language and content	(02) find out how you can receive alerts from security agencies and aid organizations about acute health hazards?	(02) herauszufinden, wie Sie Warnmeldungen von Sicherheitsbehörden und Hilfsorganisationen zu akuten Gesundheitsgefahren erhalten können?	Access	Before	Access to care
(03) find information about which health-related documents should be included in an emergency backpack (e.g., allergy pass, vaccination certificate, medication plan)?	0.75	Linguistic revision; the term “emergency backpack” has been avoided because of misunderstandings; focussed on documents	(03) find information about which health-related documents you should have ready in case of an emergency (e.g., allergy pass, vaccination certificate, medication plan)?	(03) informationen darüber zu finden, welche gesundheitsrelevanten Dokumente Sie für Katastrophenfälle bereit halten sollten (z. B. Allergiepass, Impfpass, Medikamentenplan)?	Access	Before	Prevention
(04) decide what medication or aids you need to have in stock to be able to survive for at least 2 days without outside help in the event of a crisis or disaster.	0.83	The German term “Hilfsmittel” (aids) was misleading and has been deleted from the item	(04) decide what medication you need to have to hand in order to survive for at least 2 days without outside help in the event of a disaster?	(04) zu entscheiden, was Sie an Medikamenten griffbereit haben müssen, um im Katastrophenfall mindestens zwei Tage ohne fremde Hilfe zurechtzukommen?	Apply	Before	Prevention
(05) find out where you can find protection from health hazards in the event of a crisis or disaster in your area (e.g., assembly points, contact points for the population).	0.92	No changes required	(05) find out where you can find protection from health hazards in the event of a disaster in your area (e.g., assembly points, contact points for the population)?	(05) herauszufinden, wo Sie bei einem Katastrophenfall in Ihrer Nähe Schutz vor Gesundheitsgefahren finden können (z. B. Sammelplätze, Anlaufstellen für die Bevölkerung)?	Access	Before	Access to care
(06) assess whether a health warning from a safety authority or aid organization requires immediate action on your part (e.g., evacuation call, flood risk)	0.67	Slightly linguistic revision	(06) assess whether a health warning from security authorities and civil protection organizations requires immediate action on your part (e.g., an evacuation call)?	(06) zu beurteilen, ob eine Gesundheitswarnmeldung von Sicherheitsbehörden und Hilfsorganisationen von Ihnen sofortiges Handeln erfordert (z. B. Evakuierungsaufruf)?	Appraise	During	Acute response
(07) use information to apply personal protective equipment safely (e.g., breathing masks, disposable gloves, life jackets)	0.58	Item changed to “understand”; Linguistic clarification;	(07) understand information about how to use personal protective equipment safely (e.g., breathing masks, disposable gloves, life jackets)?	(07) informationen darüber zu verstehen, wie Sie eine persönliche Schutzausrüstung sicher anwenden (z. B. Atemschutzmasken, Einmalhandschuhe, Rettungswesten)?	Understand	During	Acute response
(08) find information about where you can get help in the event of an emergency or disaster if you have health problems.	0.75	The term “emergency was removed from the item, slight linguistic adjustment”	(08) find out who to turn to for help if you have health problems in the event of a disaster?	(08) informationen dazu zu finden, bei wem Sie im Katastrophenfall Hilfe erhalten können, wenn Sie gesundheitliche Probleme haben?	Access	During	Access to care
(09) assess information about who in your area could be at risk of health problems in the event of a crisis or disaster (e.g., young children, the very old, people with visual, hearing or mobility impairments)?	0.58	Deleted because of low I-CVI and feedbacks from cognitive interviews					
(10) assess whether people around you are exposed to health risks during a crisis or disaster (e.g., due to buildings at risk of collapse, flooding, fires).	0.67	linguistic revision	(09) assess whether people around you could be at particular risk of health problems in the event of a disaster (e.g., people with visual, hearing or mobility impairments)?	(09) einzuschätzen, ob Personen in Ihrem Umfeld bei einer Katastrophe gesundheitlich besonders gefährdet sein könnten (z. B. seh-, hör-, bewegungsbeeinträchtigte Personen)?	Appraise	During	Acute response
(11) understand information on how to act if people around you are injured in a crisis or disaster (e.g., first aid)?	0.75	Item changed from “understand” to “apply”; slight linguistic adjustment	(10) to act correctly based on information if other people around you have been injured during a disaster?	(10) sich anhand von Informationen richtig zu verhalten, wenn andere Personen in Ihrem Umfeld während einer Katastrophe verletzt wurden?	Apply	During	Prevention
(12) use information about how you can support first responders and disaster relief organizations in their work during crises and disasters.	0.67	Deleted because of feedback from cognitive interviews and low relevance for the whole instrument					
(13) to use information about how to behave when water, air or food is contaminated in order to protect yourself from infections or illnesses.	0.75	Transformed to a “understand” item; slight linguistic adjustment	(11) understand what to do if water, air or food is contaminated in order to protect yourself from health hazards?	(11) informationen dazu zu verstehen, was Sie bei Verunreinigungen im Wasser, der Luft oder der Nahrung tun müssen, um sich vor Gesundheitsgefahren zu schützen?	Understand	After	Acute response
(14) find information on how to deal with health issues that may arise after a crisis or disaster (e.g., anxiety, restlessness, pain, discomfort)	0.67	Slight adjustment and shortening of the examples	(12) find information on how to deal with health issues that may arise after a disaster (e.g., discomfort, anxiety, grief)?	(12) informationen darüber zu finden, wie Sie mit gesundheitlichen Belastungen umgehen können, die nach einer Katastrophe auftreten (z. B. Unwohlsein, Angst, Trauer)?	Access	After	Access to care
(15) understand information about how to get replacements for assistive devices lost during a crisis or disaster (e.g., glasses, hearing aid, electronic reading and writing aid, wheelchair).	0.42	Deleted because of low I-CVI and feedback from cognitive interviews					

After intensive discussion in the research team and in line with the expert feedback, items 9 and 15 were removed from the item list because of their low I-CVI. It was also pointed out in the cognitive interviews that item 9 would be difficult to distinguish from item 10 and item 15 would be difficult to interpret. We carefully rephrased some other items, including item 7 (“…use information to apply personal protective equipment safely”) which we considered essential to determining DIS-HL. We no longer used terms such as “crisis” or “emergency” but instead spoke of a “disaster” throughout the instrument. Where possible, we simplified or shortened the items to make them more understandable for the general population. Wherever possible, we also avoided giving examples so as not to overly restrict the imagination of the future respondents. As a result of this process, the instrument was fundamentally revised and ultimately reduced to 12 items in combination with a short introductory text. Based on the interviewer’s feedback from the real-life interviews, the introduction had to be shortened further to avoid overwhelming respondents when the short instrument is used with the HLS_19_-Q47 questionnaire. In addition, the examples in some of the items had to be reviewed again. Overall, this part of the face validity test was successful, with all 12 items being understood and answered without any difficulty. The DIS-HL^GER^ instrument can be found in Table 3 in the original German version, as well as in an unauthorized English translation and with some details on the instrument development process.

## 4 Discussion

The main goal of this research was to develop and validate a novel instrument for assessing and monitoring the increasingly important topic of disaster-related health literacy. Our intention was to design a short and easy-to-use tool based on a conceptual framework that can be applied in the future to supplement well-established survey instruments, such as the HLS_19_ questionnaires (19, 20). Based on Sørensen et al. (19), we applied the same logic to DIS-HL as was used in the instruments developed to date for measuring topic-specific health literacy (22–24), and we also targeted the same cognitive processes as for general health literacy. The results of the content and face validation process confirms that the new DIS-HL^GER^ instrument promises to be a valuable resource to assess and monitor what we defined as the “*ability to access, understand, appraise and apply information to protect one’s own life and health or to deal with health problems before, during or after a disaster of any kind.”*

Firstly, this research highlighted the need to address the processing and handling of health information in the context *of all types of disasters* (28, 29), and thus helped to broaden the focus of the current debate in the health literacy community. Only recently, the COVID-19 pandemic with all its disruptions and physical and mental burdens, has shown how important it is for the population to have access to information about how they can protect and help themselves, especially when faced with unexpected and adverse circumstances (10, 56, 57). For this reason, some attempts have already been made to develop instruments to measure COVID-19 health literacy (25–27), and some of the authors even suggest applying them to other infectious diseases (such as Cholera, Ebola, Yellow Fever) (25). However, the discourse has so far been limited to pandemics. This overlooks the fact that comparable challenges in information processing and handling also apply to other disastrous events that are occurring with ever greater frequency and intensity, be they heat waves or other extreme weather events, earthquakes or landslides, accidents or malfunctions in technical or industrial facilities or terrorist attacks, and armed conflicts. Depending on exposure, vulnerability and capacity, all these hazards pose specific health risks, the prevention, avoidance or management of which requires the population to be able to handle targeted information (8, 10). For this reason, it would be misleading to develop separate instruments for each specific health crisis or disaster. Instead, it would be reasonable to broaden the perspective by building on the increasing discussions on disaster risk reduction, for example on “Disaster Literacy” (11) or “Disaster Risk (Reduction) Literacy” (12, 50), and to link them with the activities of the HLS-EU consortium. In this way, a well-founded contribution can be made to a topic- and context-specific differentiation of health literacy.

Secondly, this research has made it clear that efforts in the field of disaster risk reduction need to pay more attention to how and by whom health information is handled and therefore to the prerequisites of life and health protection before, during and after disasters. The fact that authorities and organizations with security responsibilities or professional helpers cannot always provide immediate assistance means that the ability to protect and help oneself is becoming increasingly important. This explains why disaster risk reduction and disaster management strategies place so much emphasis on risk communication and the public’s own preparations (1, 3, 62) – not least in Germany (2). However, these activities continue to focus on a standard population that can easily process and utilize information and has sufficient resources to help themselves before, during or after a disaster. The large number of people with reading, writing and numeracy difficulties (5) is just as often overlooked as the large proportion of the population with existing health problems or other functional or access needs (6–9). Measuring, monitoring and promoting their ability to access and handle information which helps them to protect their own life and health or to deal with health problems before, during or after a disaster of any kind is a high priority to ultimately avoid unwanted loss of life and health. At the same time, it can contribute to further developing the national disaster strategy, supporting the targeted deployment of emergency and disaster resources and preventing the overloading of critical infrastructure such as emergency centers in hospitals or community shelters in the event of a disaster (7, 8).

Thirdly, it is of high importance to notice in this context that DIS-HL is a relational concept – according to a common understanding of the health literacy concept (61, 63). A person’s actual health literacy in particular situation depends on their personal general and disaster specific health literacy, on the one hand, and on the complexity and demands of the situation (before, during or after a disaster), as well as on the disaster protection system or emergency health care system installed in the respective country, on the other. It also depends to a high degree on the form and quality of information provided by governmental or non-governmental sources to support (health-related) self-protection and self-help or to use help from disaster experts provided (10). This last aspect is of the utmost importance, especially since targeted and intended disinformation also plays a major role in connection with crises and disasters. It could even be argued that this disinformation is a disaster in itself for which one should prepare (62, 64). The information on disaster risk reduction and response should be user-friendly, but above all diversified and tailored to the different information needs of the population. This is especially true for people with functional and access needs (7, 8). Assessing and monitoring DIS-HL in the general population as well as in at-risk populations can help to increase the appropriateness and targeting of disaster-related information services. With our systematic process of instrument development, we have made a substantial contribution to this.

### 4.1 Limitations

In addition to the strengths of our research, some limitations should not go unnoticed in order to interpret the results: (1) It is possible that the scoping review was not comprehensive enough and that we overlooked publications. This could be particularly attributed to the heterogeneous terminology used in the two subject areas. However, a thorough analysis and the fact that mutual references to each other were repeatedly found in the individual publications suggest that we have covered and evaluated the essential literature on this topic. (2) Our efforts were aimed at creating a short instrument that can be used as a supplement with existing HLS_19_ questionnaires. In doing so, we were aware that the length of the instrument should be sufficiently long to measure what it is supposed to measure. It was a challenge to consider four cognitive steps, the three phases of the disaster cycle and the three areas of responsibility in this process. However, we believe that the item list, which has been carefully revised, reformulated and shortened, is a good reflection of the overall construct. Furthermore, combining the short instrument with the existing HLS_19_ questionnaires and individual items contained therein (e.g., item 3, 7 and 15 in the HLS_19_-Q47) may provide additional interesting insights. (3) The current German version of the instrument is geared toward this cultural context and the local conditions and requirements. It is important to note that structures and services in disaster risk reduction are shaped by the national context in a particular way, which is evident when looking at existing instruments. A systematic translation and cultural adaptation of the DIS-HL^GER^ instrument are still pending but can easily be carried out later. (4) Regarding the validation process reported here, it should be noted that the S-CVI of the 15-item version of the instrument (0.7) was below the recommended cut-off value (0.8). However, from the experts’ feedback, it was evident that some of them were not very familiar with either the health literacy discourse or the disaster topic. It was difficult to find experts in Germany who could cover both subject areas, which is why the number of experts involved was relatively small. A larger group of experts with knowledge of both fields might have led to a different result. For these reasons, we considered a lower S-CVI to be acceptable. Furthermore, thanks to the experts’ valuable suggestions, we were able to eliminate items with a very low I-CVI, shorten the instrument to 12 items, adjust the content and wording of critical items and improve the introductory text. The encouraging results of the face validity test also helped and supported the successful development of the DIS-HL^GER^ instrument. (5) The inclusion of population groups with a low level of education or functional needs in the context of promoting health literacy in disasters is of central importance. It is therefore also important that their specific requirements for the accessibility of the measurement instrument are carefully considered. This aspect was analyzed as part of the validation and taken into account by adapting the items (e.g., in terms of their comprehensibility). Nevertheless, the accessibility criteria should be carefully observed and reflected upon during the piloting and future use of the instrument. (6) This article is limited to the documentation of the development and conceptualisation of the DIS-HL^GER^ instrument and its testing for content validity and face validity. Subsequently, a pilot test and psychometric testing of the instrument was carried out as part of the third national representative survey on health literacy in Germany (HLS-GER 3). The procedure and results of this phase of instrument development, in which the authors of this paper were involved, will be analyzed and documented in a further publication. Based on these results, an English translation and international adaptation of this instrument could be considered. However, the strong roots of the DIS-HL^GER^ instrument in the German context should be carefully considered in this cross-cultural adaptation.

## 5 Conclusion

Numerous disasters, including pandemics, floods, droughts and heat waves, terrorist attacks and armed conflicts, have occurred in many parts of the world, including Europe, in recent times, resulting not only in significant material damage but also in considerable health impacts and even loss of life. There is no doubt that it is important to prepare better and more effectively for such events in the future and that, in addition to other measures, the ability of the population to protect and help themselves when faced with adversity must be strengthened. Therefore, the *“ability to access, understand, appraise and apply information that is geared towards this self-protection and self-help before, during and after disasters of all kinds”* is of central importance. However, little research has been done to date on the level of this so-called DIS-HL in the population or in selected population groups. Previous activities are either too topic- or context-specific and not focused enough on health issues. Available instruments to measure disaster or disaster risk literacy are either too long, insufficiently substantiated, not translated or not compatible with the activities of the HLS-EU Consortium and its understanding of health literacy and its measurement. The lack of suitable instruments and thus also of corresponding information on the level of DIS-HL in the population or selected population groups makes it difficult to develop targeted information to be used in health and disaster risk education. The short DIS-HL^GER^ measurement instrument, developed in this research, is based on a conceptual framework, validated and promises to be relevant, meaningful and easy to use in combination with existing HLS_19_ questionnaires. It thus fills an important research gap. However, continuously review will be necessary to further evaluate the validity of this tool and to improve it systematically. This upcoming research should not only further improve the quality of the instrument but also stimulate international debate on the important topic of disaster-related health literacy.

## Data Availability

The datasets presented in this article are not readily available because the participants of this study (expert review, cognitive interviews and real-life interviews) did not give written consent for their data to be shared publicly. Requests to access the datasets should be directed to michael.ewers@charite.de.
