# Correction: Disaster health literacy – development and validation of a short measurement instrument in German to supplement the HLS_19_ instruments

**DOI:** 10.3389/fpubh.2025.1691501

**Published:** 2025-10-03

**Authors:** Michael Ewers, Joachim Beckert, Lennert Griese, Michael Köhler, Anita Prasser, Himal Singh, Doris Schaeffer

**Affiliations:** ^1^Institute of Health and Nursing Science, Charité - Universitätsmedizin Berlin, Corporate Member of Freie Universität Berlin and Humboldt-Universität zu Berlin, Berlin, Germany; ^2^School of Public Health, Bielefeld University, Bielefeld, Germany

**Keywords:** disaster & risk management, health literacy, instrument development, measurement, content validity, face validity, disaster health literacy

The definition of “Disaster Literacy” from reference “Çalişkan, C, and Üner, S. Measurement of disaster literacy in Turkish society: disaster literacy scale (DLS) design and development process. *Disaster Med Public Health Prep*. (2023) 17:e211. doi: 10.1017/dmp.2022.147” was not fully or correctly quoted. In addition, the authors incorrectly stated the number of items in Çalişkan's instrument (Disaster Literacy Scale). It previously stated “84” (before validation), but there are actually “61” items (after validation).

A correction has been made to the section [3. Results, *3.1 Scoping Review, paragraph 5*]:

“By contrast, Çalişkan et al. [11] defined “Disaster Literacy” based on a systematic literature review as an “individuals' capacity to access, understand, appraise, and apply disaster information to make informed decisions and to follow instructions in everyday life concerning mitigating/prevention, preparing, responding, and recovering/rehabilitation from a disaster in order to maintain or improve quality of life during the life course” [34] (p. 2). Based on this definition they developed a complex 16-Matrix integrative conceptual model and a self-report measurement tool for Turkey, the so-called “Disaster Literacy Scale” (DLS). This tried-and-tested instrument has 61 items and has, to our knowledge, so far been used with different populations but only in this specific cultural context [34, 55, 56].”

The original version of this article has been updated.

